# Dopamine D_3_ Receptors Inhibit Hippocampal Gamma Oscillations by Disturbing CA3 Pyramidal Cell Firing Synchrony

**DOI:** 10.3389/fphar.2015.00297

**Published:** 2016-01-06

**Authors:** Clément E. Lemercier, Steffen B. Schulz, Karin E. Heidmann, Richard Kovács, Zoltan Gerevich

**Affiliations:** Institute of Neurophysiology, Charité - Universitätsmedizin BerlinBerlin, Germany

**Keywords:** gamma rhythm, dopamine D_3_ receptors, hippocampus CA3 region, cognition, schizophrenia, antipsychotics

## Abstract

Cortical gamma oscillations are associated with cognitive processes and are altered in several neuropsychiatric conditions such as schizophrenia and Alzheimer’s disease. Since dopamine D_3_ receptors are possible targets in treatment of these conditions, it is of great importance to understand their role in modulation of gamma oscillations. The effect of D_3_ receptors on gamma oscillations and the underlying cellular mechanisms were investigated by extracellular local field potential and simultaneous intracellular sharp micro-electrode recordings in the CA3 region of the hippocampus *in vitro*. D_3_ receptors decreased the power and broadened the bandwidth of gamma oscillations induced by acetylcholine or kainate. Blockade of the D_3_ receptors resulted in faster synchronization of the oscillations, suggesting that endogenous dopamine in the hippocampus slows down the dynamics of gamma oscillations by activation of D_3_ receptors. Investigating the underlying cellular mechanisms for these effects showed that D_3_ receptor activation decreased the rate of action potentials (APs) during gamma oscillations and reduced the precision of the AP phase coupling to the gamma cycle in CA3 pyramidal cells. The results may offer an explanation how selective activation of D_3_ receptors may impair cognition and how, in converse, D_3_ antagonists may exert pro-cognitive and antipsychotic effects.

## Introduction

Cognitive impairment is common in individuals with neuropsychiatric disorders such as schizophrenia, mood disorders, Alzheimer’s disease, autism spectrum disorder, and Parkinson’s disease (Millan et al., 2012). Many efforts have been made to develop drugs to maintain or enhance cognitive processes but the available treatments still have limited pro-cognitive effects. Despite some progress in treating cognitive deficits in schizophrenia with second-generation antipsychotics, management of negative and cognitive symptoms remains one of the most pressing and unresolved problems of neuropsychopharmacology (Miyamoto et al., 2012).

In recent years, there has been increasing recognition of the role of D_3_ receptors in cognition (Nakajima et al., 2013). Behavioral studies in a variety of animals have shown that activation of D_3_ receptors impairs attention, working memory, object recognition, associative learning, episodic memory, and spatial learning (Ukai et al., 1997; Smith et al., 1999; Watson et al., 2012), whereas blockade or knockout of this receptor seems to exert pro-cognitive effects (Glickstein et al., 2005; Laszy et al., 2005; Loiseau and Millan, 2009; Xing et al., 2010). Accordingly, in humans, activation of these receptors has been shown to impair cognitive performance, whereas their blockade seems to have pro-cognitive effects (Cools et al., 2006; Hamidovic et al., 2008; Gross et al., 2013). Despite the increasing evidence for D_3_ receptor involvement on cognitive processes, the underlying mechanism is still not understood (Nakajima et al., 2013).

Gamma band (30–90 Hz) oscillations have been implicated in a range of aforementioned cognitive processes (Axmacher et al., 2010; Powell et al., 2014) and are known to be generated by the synchronous firing of perisomatic parvalbumin containing fast-spiking basket cells (Gulyás et al., 2010). An increasing number of studies have demonstrated that gamma oscillations are altered and instable in schizophrenic patients and have led to the hypothesis that disturbances in gamma band network activity may be involved in the pathophysiology of the disease (Uhlhaas and Singer, 2010). Associations between gamma disturbances and positive, negative and cognitive symptoms have been observed (Light et al., 2006; Lee et al., 2010; Mulert et al., 2011) making gamma rhythm abnormalities an emerging biomarker in schizophrenia with a potential for use in drug development. In our previous study on the effects of first and second generation antipsychotics on hippocampal gamma oscillations (Schulz et al., 2012a) we found that among 19 investigated receptors, the 5-HT_3_ and D_3_ receptors seemed to be most strongly implicated in the effect of antipsychotics on hippocampal gamma oscillations. Indeed, elevated D_3_ receptor expression have been found in the central nervous system of schizophrenic patients (Gurevich et al., 1997). Moreover, D_3_ receptor polymorphisms have been reported to be associated with schizophrenia (Jönsson et al., 2003; Talkowski et al., 2006) and poorer working memory and executive functioning tasks (Szekeres et al., 2004).

In present study, we investigated the effect of D_3_ receptors on hippocampal gamma oscillations and its underlying cellular mechanisms. Our findings show that D_3_ receptor activation decreases the power, coherence and dynamics of gamma oscillations and that this effect is accompanied by a reduction of the firing rate in CA3 pyramidal cells and the synchrony of their spiking within the gamma cycle. In light of the relationship between hippocampal gamma rhythms and higher cognitive functions, our data offer an explanation how D_3_ receptor activation may exert anti-cognitive action and how its blockade may have pro-cognitive and antipsychotic effects.

## Materials and Methods

### Slice Preparation

Hippocampal slices were prepared from Wistar rats of both sexes at an age of 6–9 weeks (180–230 g). Animal procedures were conducted in accordance with the guidelines of the European Communities Council and the institutional guidelines approved by the Berlin Animal Ethics Committee (Landesamt für Gesundheit und Soziales Berlin, T0330/12). All efforts were made to minimize animal suffering and to reduce the number of animals used. Animals were anesthetized with isoflurane and then decapitated. Their brains were removed and immerged in ice-cold artificial cerebrospinal fluid (ACSF; in mM: NaCl, 129; KCl, 3; NaHCO_3_, 21; NaH_2_PO_4_, 1.25; MgSO_4_, 1.8; CaCl_2_, 1.6; glucose, 10) aerated with carbogen gas (95% O_2_/5% CO_2_). The brain was cut into 400 μm thick horizontal slices containing the hippocampal formation with a vibratome (DSK microslicer DTK-1000, Dosaka, Japan). Slices were immediately transferred to an interface-type recording chamber perfused with warm and carbogenated ACSF (36°C, flow rate 1.7 ml/min). Slices were left for recovery for at least 1 h before starting the experiments.

### Extracellular Recordings

Local field potentials were recorded from stratum pyramidale of area CA3b with glass pipettes filled with ACSF (resistance < 3 MΩ) as earlier described (Klaft et al., 2012; Çalişkan et al., 2015). Recordings were amplified by a custom-made amplifier, low-pass filtered at 1 kHz and sampled at 5 kHz by a CED 1401 interface (Cambridge Electronic Design, Cambridge, UK). Gamma oscillations were induced by bath application of either 10 mM acetylcholine (ACh) and 2 mM physostigmine (Physo) or 100 nM kainic acid (KA) and stabilized after 90 and 50 min, respectively. Drugs were applied 100 and 60 min after the application of ACh/Physo and KA, respectively, for a period of 60 min. Antagonists, if appropriate, were applied 40 min prior to the agonist or the wash out of ACh/Physo. Note that for the better oxygen supply in the tissue, gamma oscillations were evoked in an interface-type chamber showing slower equilibration of slices with drugs than in submerged chambers (Hájos et al., 2009).

### Intracellular Recordings

Intracellular recordings were made after the induction of ACh-induced gamma oscillations from CA3b pyramidal cells in the slice with sharp microelectrodes filled with 2 M K^+^-acetate (resistance 70.3 ± 5.4 MΩ) as described earlier (Schulz et al., 2012b). Intracellular signals were amplified by a SEC-05 LX amplifier (npi electronics, Tamm, Germany), low-pass filtered at 2 kHz and sampled at 10 kHz using the CED 1401 interface. Recordings were done in bridge mode. Cells were impaled during the induction of gamma oscillations. The measurements were started after the stabilization of gamma oscillations but at least 20 min after penetration. Only cells were accepted which showed stable overshooting APs over the full period of the experiment.

### Materials

KA, Physo, PD-128907, PG-01037, and L-741,626 were purchased from Tocris Bioscience (Bristol, UK). ACh was purchased from Sigma–Aldrich (Taufkirchen, Germany).

### Data Analysis and Statistics

For the analysis of oscillations, power spectra were calculated every 2 min with a 2-min window throughout the recording and peak power, peak frequency and half bandwidth (at 50% of peak power) were determined off-line by using a custom-made script for the Spike2 software (Cambridge Electronic Design, Cambridge, UK). Since absolute power values vary substantially among slices, they were normalized in every slice to a 10-min period before the drug application, the ACh/Physo washout or the corresponding time in control experiments. Data are presented as mean ± SEM. Statistical comparisons between the drug-induced effects and the time-matched control experiments were made using Student’s *t*-test. Significance level was set at *p* < 0.05.

Phase histograms of APs from intracellular recordings in relationship to the extracellular gamma cycle and the corresponding LFP waveform averages were calculated by the Spike2 software over time windows covering 1000 APs each. 0° represents the trough of the LFP gamma cycles. Occurrence of fast components at the negative peak of gamma oscillations (most probably spikes in pyramidal cells adjacent to the electrode tip) made a low-pass filtering (100 Hz) of the data necessary (Fisahn et al., 1998). Although the FIR filter preserves the shape and phase of the signal better than the infinite impulse response (IIR) filter, we observed a minimal ∼10° shift in phase which, however, did not bias the calculated changes in phase accuracy or preferred phase induced by drugs. Analysis of the phasic AP timing resulted in a mean vector for each cell. Its mean phase Φ and vector lengths *r* were used to calculate the mean vectors and the circular standard deviations for the cell populations of different drug conditions (Schulz et al., 2012b). Time-frequency-analysis of LFPs was carried out oﬄine using the Spike2 software.

## Results

### Dopamine Inhibits Cholinergically Induced Gamma Oscillations in the Hippocampus

Perfusion of the hippocampal slices with acetylcholine (ACh, 10 μM) and Physo (2 μM) induced gamma oscillations in the CA3 pyramidal layer with a peak power of 429.22 ± 253.26 μV^2^, a peak frequency of 37.6 ± 0.71 Hz and a half bandwidth of 3.92 ± 0.82 Hz. A narrow half bandwidth in the power spectrum of the oscillation indicates a high temporal coherence and regular oscillations, whereas a wide gamma band means low coherence and less regular oscillations.

**FIGURE 1:**
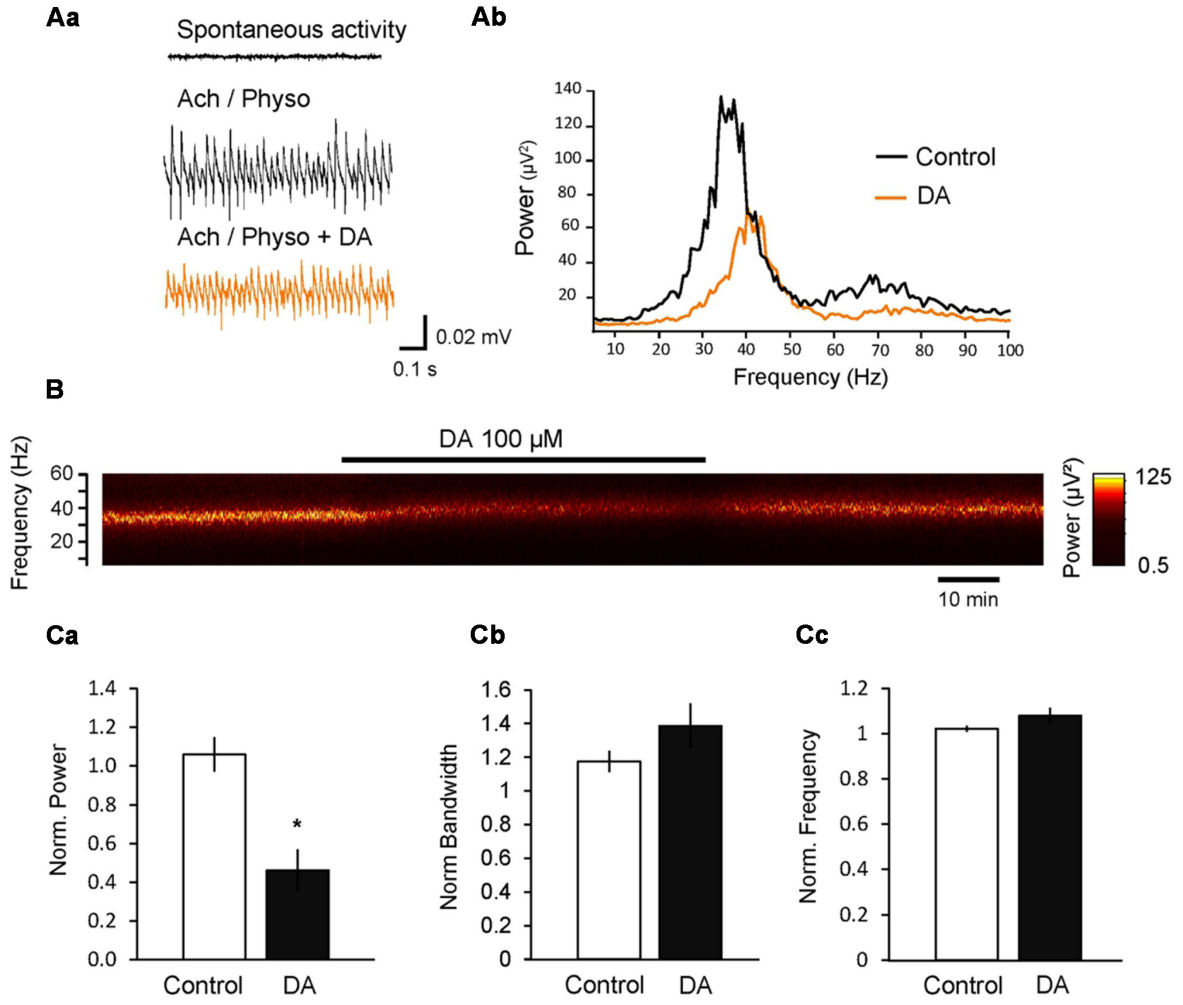
**Dopamine (DA) inhibits cholinergically induced hippocampal gamma oscillations. (Aa)** Local field potentials (LFPs) recorded in the CA3 pyramidal layer from rat hippocampal slices. Gamma oscillations were induced by bath application of acetylcholine (ACh, 10 μM) and physostigmine (Physo, 2 μM). Addition of DA (100 μM) to the bath inhibited the power of gamma oscillations. **(Ab)** Power spectra of ACh-induced gamma oscillations before and after DA application. **(B)** Spectrogram of the gamma oscillations before, during and after the application of DA. **(C)** Bars summarize the effect of DA on the peak power **(Ca)**, half bandwidth **(Cb)** and peak frequency **(Cc)**. Data were normalized to the mean of the 10-min period before DA application or the corresponding time in control experiments. ^∗^*p* < 0.05 compared to the time-matched control.

Application of dopamine (DA; 100 μM) decreased the power to 46.2 ± 10.8% (*n* = 6; *p* < 0.001, compared to control power change to 106.1 ± 8.9%, *n* = 12; **Figure 1**), whereas the peak frequency did not change significantly (108.0 ± 3.5%, *p* = 0.057 compared to control frequency change to 102.2 ± 1.0%; **Figure 1**). We also investigated the effect of DA on the width of the gamma band (half bandwidth) and found that DA did not affect significantly the bandwidth of the oscillation (138.7 ± 14.1%, *p* = 0.123 compared to control half bandwidth change to 117.4 ± 6.1%; **Figure 1**).

### Dopamine D_3_ Receptors Inhibit Cholinergically Induced Gamma Oscillations in the Hippocampus

**FIGURE 2:**
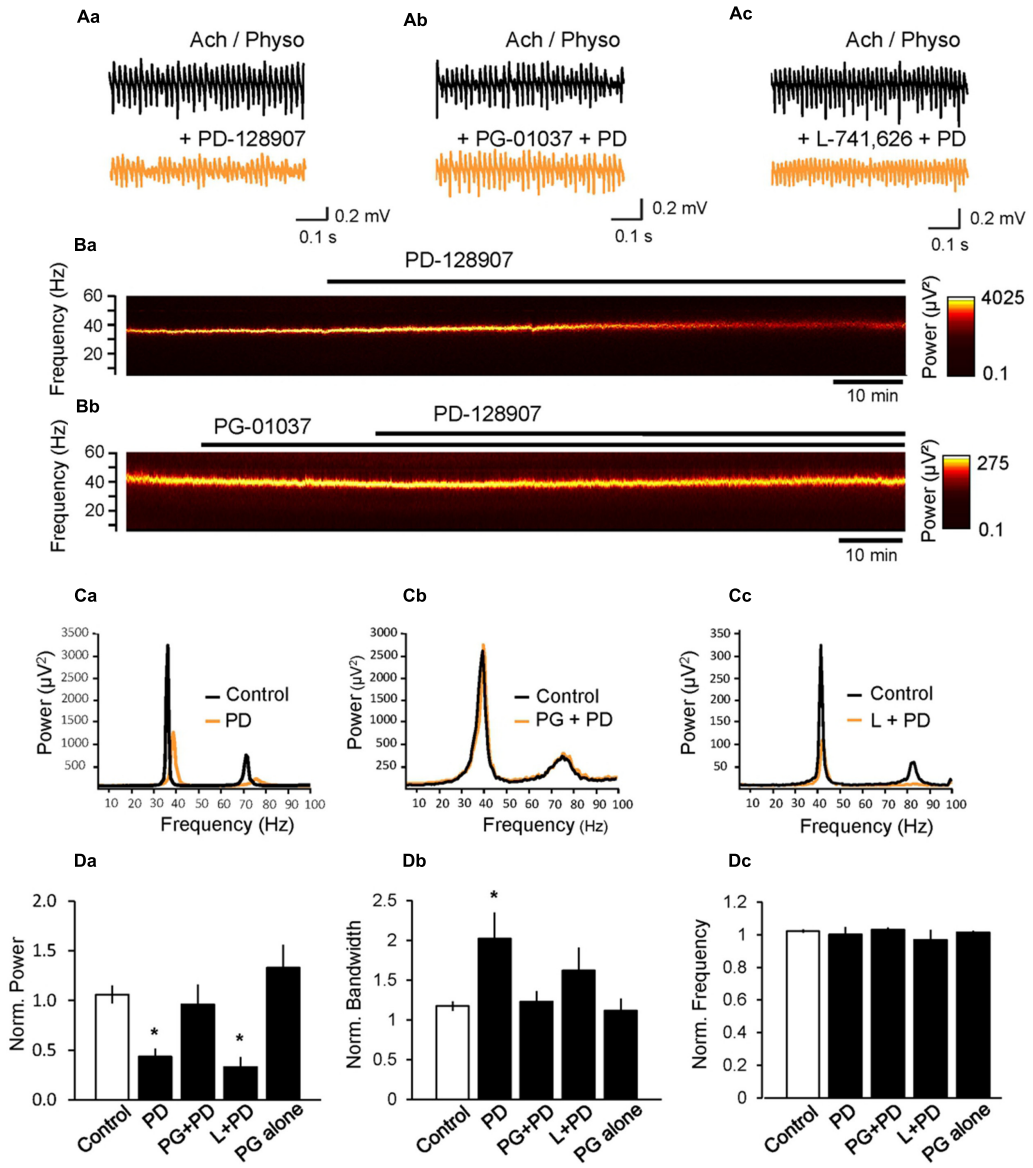
**Activation of D_3_ dopamine receptors inhibits cholinergically induced hippocampal gamma oscillations. (A)** LFP recordings from the CA3 pyramidal cell layer after induction of gamma oscillations by ACh (10 μM) and Physo (2 μM) before (black) and after (orange) the application of the selective D_3_ receptor agonist PD-128907 (PD, 10 μM) alone **(Aa)**, in the presence of the D_3_ receptor antagonist PG-01037 (10 μM, **Ab**) or in the presence of the selective D_2_ receptor antagonist L-741,626 (10 μM, **Ac**). Only the D_3_ antagonist antagonized the effect of PD-128907 indicating that only the D_3_ receptors are involved in the effect. **(B)** Spectrograms showing the effect of PD-128907 on hippocampal gamma oscillations in the absence **(Ba)** and presence **(Bb)** of the D_3_ antagonist PG-01037. PG-01037 could antagonize the effect of PD-128907. **(C)** Power spectra showing the effect of PD-128907 (PD) on hippocampal gamma oscillations alone **(Ca)**, in the presence of PG-01037 (PG; **Cb**) and in the presence of L-741,626 **(Cc)**. **(D)** Bars summarize the effect of PD-128907 alone and in the presence of selective DA receptor antagonists on the peak power **(Da)**, half bandwidth **(Db)** and peak frequency **(Dc)**. The last bar shows the effect of PG-01037 alone. Data were normalized to the mean of the 10-min period before PD-128907 application or the corresponding time in control experiments. ^∗^*p* < 0.05 compared to the time-matched control.

Our previous study on the effects of antipsychotics on gamma oscillations suggested that among DA receptors, only the activation of D_3_ receptor altered gamma oscillations significantly (Schulz et al., 2012a). To further investigate the effect of these receptors on gamma oscillations, we next applied PD-128907 (10 μM), a selective DA D_3_ agonist and found that it decreased the power to 43.4 ± 8.1% (*n* = 8, *p* < 0.001 compared to control; **Figure 2**) and broadened the half bandwidth of the gamma oscillations to 216.6 ± 37.0% (*p* = 0.003, compared to control; **Figure 2**). The peak frequency of the oscillation did not change (100.2 ± 4.9%, *p* = 0.642 compared to control; **Figure 2**). To confirm whether indeed the D_3_ receptors are responsible for the effect of PD-128907, we repeated the experiments in the presence of PG-01037 (10 μM), a selective antagonist at D_3_ receptors. PG-01037 itself did not significantly alter gamma oscillations (power: 133.0 ± 22.9%, *p* = 0.212; bandwidth: 113.9 ± 12.8%, *p* = 0.780; frequency: 101.1 ± 1.0%; *n* = 7; *p* = 0.475 compared to control; **Figure 2D**) but antagonized the effect of PD-128907 on power (96.0 ± 20.1%, *n* = 7, *p* = 0.024 compared to PD-128907 alone, **Figure 2**) and half bandwidth (123.0 ± 14.3%, *p* = 0.047 compared to PD-128907 alone, **Figure 2**), whereas the frequency of the oscillation was not changed (103.1 ± 14.7%, *p* = 0.607 compared to PD-128907 alone, **Figure 2**). On the contrary, L-741,626 (10 μM), a selective D_2_ receptor antagonist, did not reverse the effect of PD-128907 (power: 33.0 ± 9.8%, *n* = 7, *p* = 0.422; half bandwidth: 162.0 ± 31.3%, *p* = 0.199; frequency: 96.8 ± 6.5%, *p* = 0.543 compared to PD-128907 alone). These data indicate that PD-128907 reduced the magnitude and precision of gamma oscillations via selective activation of D_3_ receptors.

### D_3_ Receptors Inhibit Kainate-Induced Hippocampal Gamma Oscillations

**FIGURE 3:**
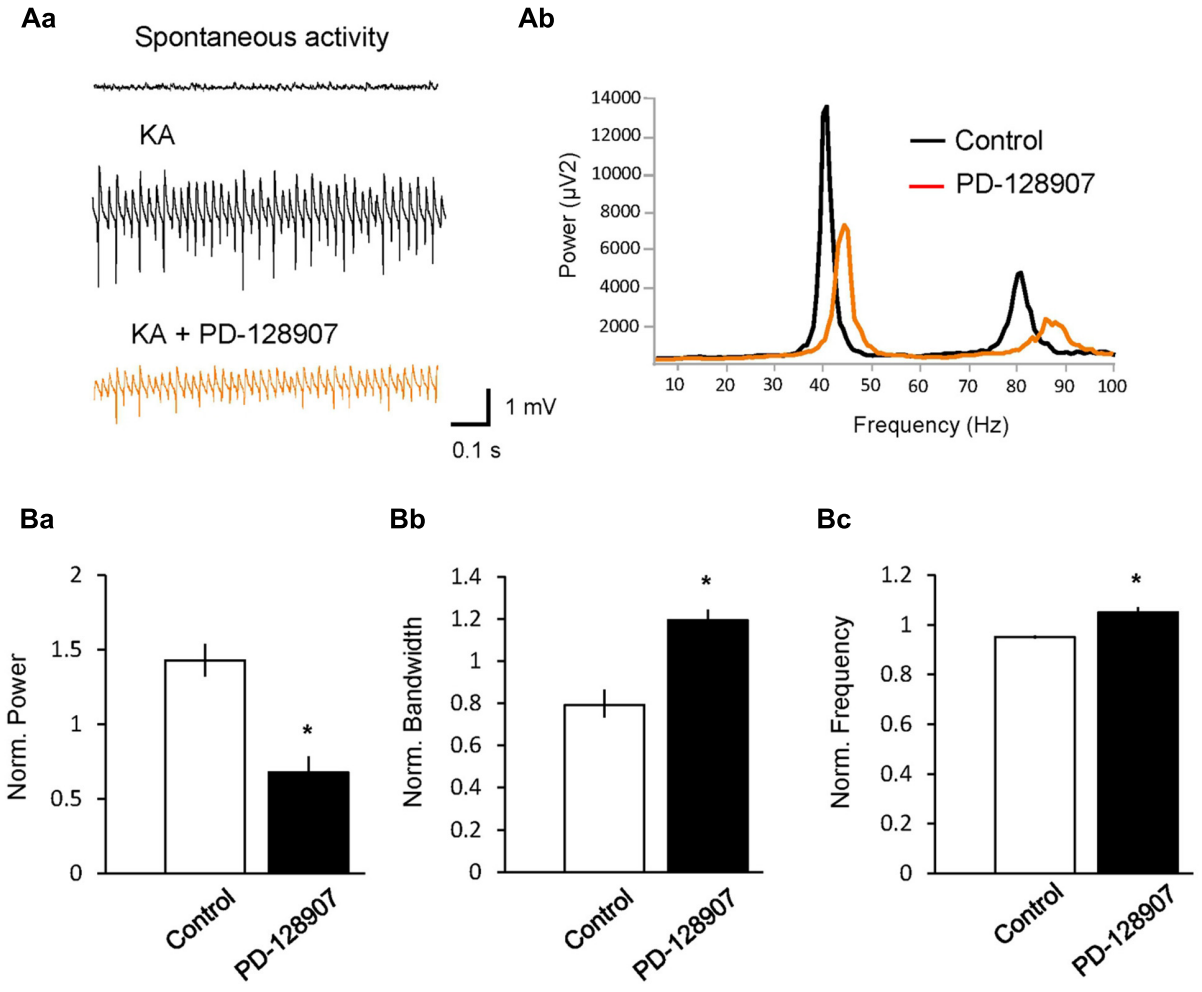
**Activation of dopamine D_3_ receptors (D_3_ receptors) inhibits kainate (KA)-induced hippocampal gamma oscillations. (A)** LFPs recorded in the CA3 pyramidal layer from rat hippocampal slices. **(Aa)** Gamma oscillations were induced by bath application of KA (100 nM). The selective D_3_ receptor agonist PD-128907 (10 μM) inhibited the power of gamma oscillations. **(Ab)** Power spectra of KA-induced gamma oscillations in the absence (black) and presence (orange) of PD-128907. **(B)** Bars summarize the effect of PD-128907 on the peak power **(Ba)**, half bandwidth **(Bb)** and peak frequency **(Bc)** of gamma oscillations. Data were normalized to the mean of the 10-min period before PD-128907 application or the corresponding time in control experiments. ^∗^*p* < 0.05 compared to the time-matched control.

Gamma oscillations can also be induced by activation of KA receptors on pyramidal and basket cells (Fisahn et al., 2004), showing a different pharmacological profile compared to ACh-induced gamma oscillations (Schulz et al., 2012b). To test whether D_3_ receptors can also modulate KA-induced gamma oscillations, we applied KA (100 nM) on hippocampal slices and reliably induced gamma oscillations in the CA3 pyramidal layer with a peak power of 943.31 ± 388.64 μV^2^, a peak frequency of 42.72 ± 0.96 Hz and a half bandwidth of 3.72 ± 0.85 Hz. Application of PD-128907 decreased the power to 67.7 ± 11.9% (*n* = 5; compared to KA control: 142.9 ± 12.2%, *n* = 5; *p* = 0.002) and broadened the bandwidth to 119.4 ± 5.7% (compared to KA control: 79.2 ± 8.2; *p* = 0.004; **Figure 3**). The frequency increased to 105.0 ± 1.8% (compared to KA control: 95.2 ± 0.6%; *p* = 0.001). We compared the effects of PD-128907 on ACh- and KA-induced gamma oscillations in **Table 1**. As seen, we did not find any statistical different effects on any parameters between the two induction protocols.

**Table 1:** Effects of PD-128907 on ACh- and KA-induced gamma oscillations.

Oscillation parameter	ACh (10 μM) + Physo (2 μM)	KA (100 nM)
Peak power (mV^2^)	43.4 ± 8.1%*	67.7 ± 11.9%*
Peak frequency (Hz)	100.2 ± 4.9%	105.0 ± 1.8%*
Half bandwidth (Hz)	216.6 ± 37.0%*	119.4 ± 5.7%*

*Data were normalized to the baseline period before PD-128907 application. ^∗^*p* < 0.05 compared to control experiment.*

### D_3_ Receptors Slow Down the Development of Gamma Oscillations

**FIGURE 4:**
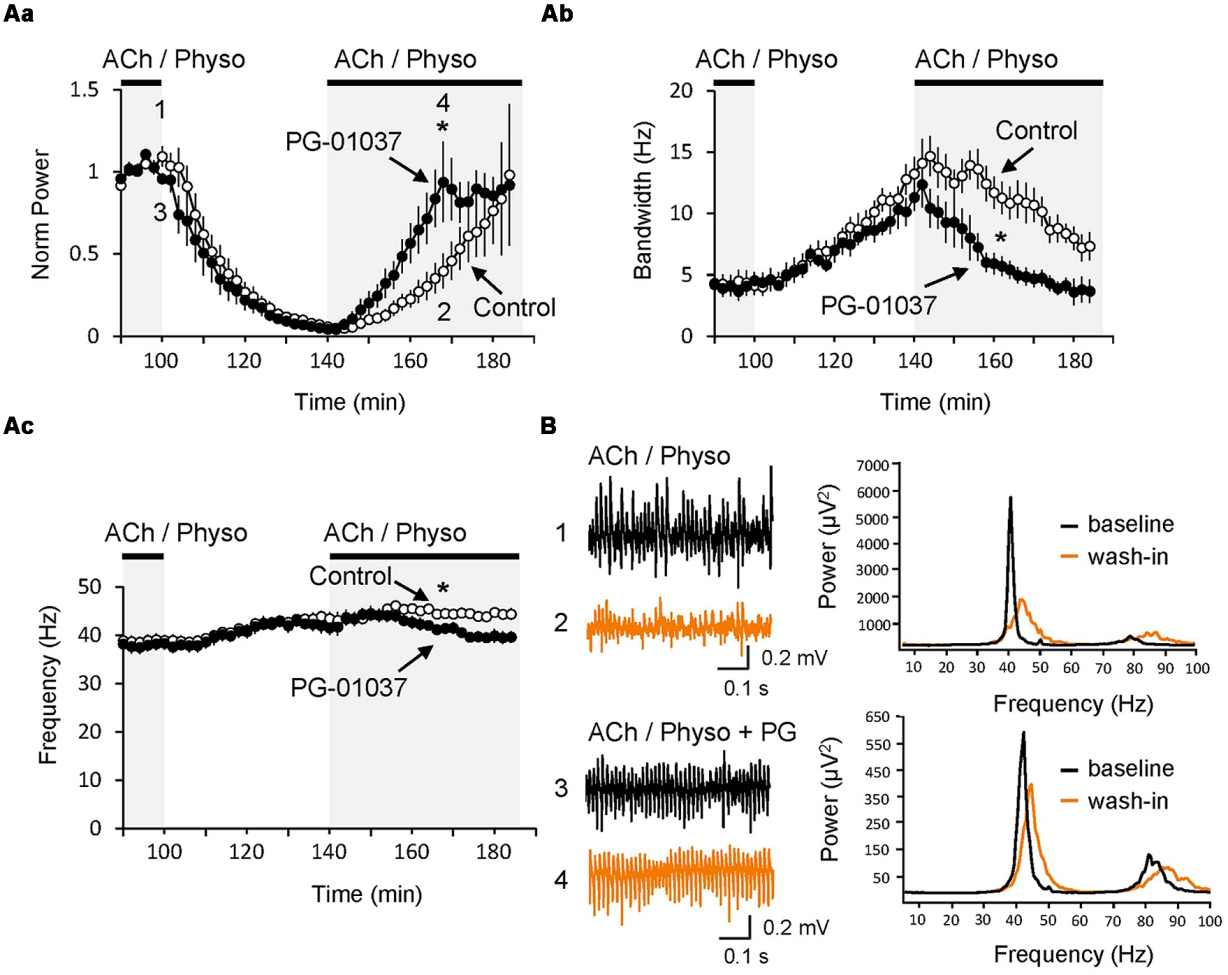
**Inhibition of D_3_ receptors speeds re-synchronization of hippocampal gamma oscillations. (A)** The selective D_3_ receptor antagonist PG-01037 (10 μM, black circles) did not affect the decay of gamma oscillations after the washout of ACh and Physo but significantly fastened their re-synchronization as seen in the faster increase of the power **(Aa)**, faster narrowing of the half bandwidth **(Ab)** and reversing of the original peak frequency **(Ac)**. In control experiments no PG-01037 was applied (open circles) (**B;** Left) LFP recordings before washing out of ACh and Physo (black) and after the re-perfusion of the same drugs (orange). (Right) Power spectra for the same recordings marked with the same colors. ^∗^*p* < 0.05 compared to the time-matched control.

Since *in vivo* cortical network activity is dynamic and characterized by appearing and disappearing synchronization, we next investigated whether D_3_ receptors could have an effect on these dynamics. To do so, gamma oscillations were first induced by ACh and Physo (baseline state) and then washed out after stabilization of the oscillations either in the presence or the absence (control) of the D_3_ antagonist PG-01037. As a consequence of the removal of the ACh and Physo, the gamma oscillations became progressively less powerful, faster and less synchronized (**Figure 4A**). After 40 min of washout of ACh and Physo (wash-out state), the gamma power showed massive reduction (5.8 ± 1.6% of the baseline state, *n* = 9; *p* < 0.001 compared to baseline state), and both the frequency (43.4 ± 1.4 Hz; *p* = 0.013 compared to baseline state 38.9 ± 1.1 Hz) and the half bandwidth (13.2 ± 1.4 Hz; *p* = 0.010 compared to baseline state 4.1 ± 0.8 Hz; **Figure 4A**) increased. Blockade of the D_3_ receptors by PG-01037 (10 μM) did not affect the desynchronisation of the gamma oscillations (power: 4.3 ± 1.4% of the baseline state, *n* = 7, *p* = 0.497 compared to the control washout state; frequency: 41.5 ± 1.4 Hz, *p* = 0.368; bandwidth: 11.3 ± 1.1 Hz, *p* = 0.340; **Figure 4**). Next, ACh and Physo were re-perfused to the ACSF to investigate the dynamics of neuronal network resynchronization. In the control experiment, 30 min after the renewed presence of ACh and Physo (re-wash-in state), the power increased significantly to 39.6 ± 10.56% of baseline (*p* = 0.009 compared to wash-out state) and the half bandwidth started to decrease to 10.9 ± 1.6 Hz (*p* = 0.232 compared to wash-out state; **Figures 4Aa,b**). In contrast, the frequency in the re-wash-in state was unchanged when compared to wash-out state (44.5 ± 1.1 Hz; *p* = 0.497 compared to wash-out state; **Figure 4Ac**).

Interestingly, PG-01037 strongly facilitated the re-synchronization of the oscillations (**Figure 4**). 30 min after wash-in of ACh and Physo (re-wash-in state), the gamma power and the half bandwidth returned back to values comparable to those initially present during the baseline state (power: 93.7 ± 24.4% of baseline, *p* = 0.044 compared to control experiments; half bandwidth: 4.9 ± 0.7 Hz compared to the baseline state 4.0 ± 0.8 Hz, *p* = 0.012 compared to the control experiments; **Figures 4Aa,b**). Also the frequency decreased, but failed to recover to values of the baseline state. However, compared to the control group at the same time, it is significantly reduced (41.1 ± 1.0 Hz compared to control group 44.3 ± 0.9 Hz, *p* = 0.031; **Figure 4Ac**).

### D_3_ Receptors Alter Firing Properties of CA3 Pyramidal Cells

To investigate the mechanisms underlying the gamma oscillation inhibition by activation of D_3_ receptors, we recorded membrane potential from CA3 pyramidal cells by means of intracellular sharp electrodes after stabilization of cholinergic gamma oscillations. During gamma oscillations, a robust membrane potential oscillation was observed with a mean frequency of 30.2 ± 2.5 Hz and a peak-to-peak amplitude of 1.78 ± 0.57 mV. The frequency of these membrane potential oscillations was comparable to the frequency of the LFP oscillations measured in parallel by the extracellular electrode (LFP: 33.5 ± 1.57 Hz; *p* = 0.292). Application of the D_3_ receptor agonist PD-128907 reduced the peak-to-peak amplitude of the membrane potential oscillations to 0.86 ± 0.26 mV (*p* = 0.047), whereas their frequency was not changed (29.1 ± 2.8 Hz, *p* = 0.424).

The cells fired APs at a rate of 9.8 ± 2.4 Hz (*n* = 7) phase-locked to the gamma oscillations (Rayleigh test, *p* < 0.001 for each cell; Moore’s test, *p* < 0.05 for the cell population, *n* = 7). Circular analysis of APs related to the gamma cycle revealed a mean phase Φ of 15.3 ± 15.3° (mean ± circular standard error; 0° = trough of the gamma cycle) and a mean vector length r of 0.71 ± 0.07 after low-pass filtering, indicating a rather high accuracy of neuronal firing within the gamma cycle (*r* = 1 would mean that all cells fired all APs at the very same phase with maximal synchrony, *r* = 0 that all APs were equally distributed over the gamma cycle).

**FIGURE 5:**
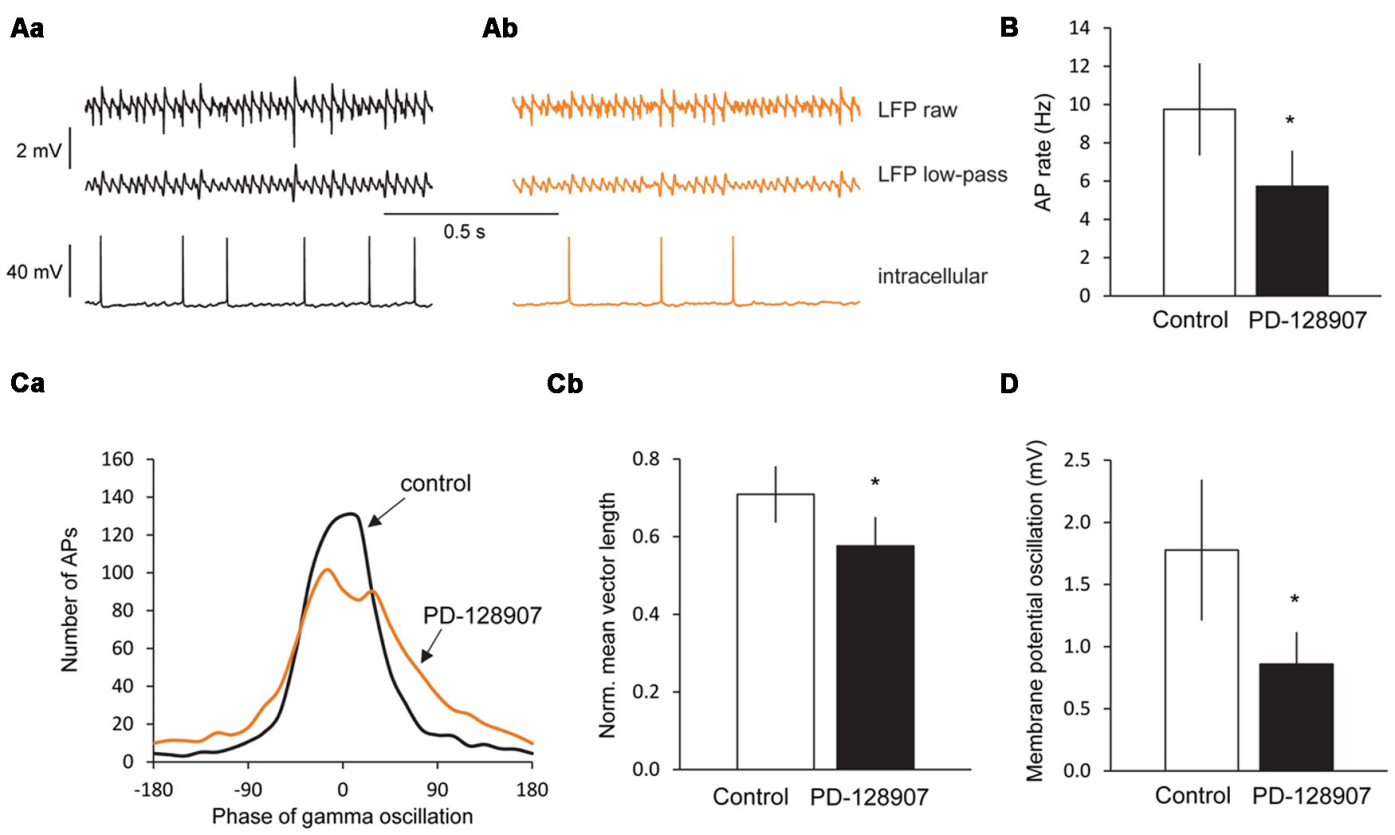
**Dopamine D_3_ receptors decrease the firing rate, firing synchrony and membrane potential oscillations in CA3 pyramidal cells during gamma oscillations. (Aa)** LFP recordings from the CA3 pyramidal cell layer (top), the low-pass (100 Hz) filtered LFP signal (middle) and the corresponding intracellular recording from a CA3 pyramidal cell (bottom) after the induction of gamma oscillations by ACh and Physo. **(Ab)** Effects of the selective D_3_ receptor agonist PD-128907 (10 μM) on the LFP recordings (top) and the corresponding intracellular recordings from a CA3 pyramidal cell (bottom). **(B)** Bars summarize the firing rates before and after PD-128907 application. **(Ca)** Phase histograms of action potentials (APs) before and after PD-128907 application. The average curve for all measured cells is shown (*n* = 7). In every cell, 1000 APs were analyzed. 0° represents the troughs of the gamma cycles after low-pass filtering. PD-128907 broadened the distribution of APs during the gamma cycle. **(Cb)** Bars summarize the mean vector lengths (*r*) before and after PD-128907 application. *r* = 1 would mean that all cells fired all APs at the very same phase with maximal synchrony; *r* = 0 that all APs were equally distributed over the gamma cycle. **(D)** Bars summarize the peak-to-peak amplitude of membrane potential oscillations from CA3 pyramidal cells before and after PD-128907 application. ^∗^*p* < 0.05 compared to the 10-min baseline period before PD-128907.

PD-128907 reduced the firing rate of pyramidal cells to 5.7 ± 1.9 Hz (*p* = 0.045, *n* = 7). Whereas Φ did not change (17.3 ± 18.4°, *p* > 0.05) indicating that the cells still fired at the same phase within the gamma cycle on average, r decreased to 0.58 ± 0.07 (*p* = 0.027, Hotelling test for paired samples; **Figure 5**) suggesting that the synchrony of spike timing was reduced after D_3_ receptor activation.

Analyzing spontaneous APs during gamma oscillations before and after PD-128907 application revealed that neither the AP half-width (control: 1.02 ± 0.04 ms; PD-128907: 1.04 ± 0.05; *p* = 0.680, not shown) nor the AP amplitude (control: 54.0 ± 3.2 mV; PD-128907: 54.1 ± 4.4 mV; *p* = 0.978, not shown) were affected.

## Discussion

### D_3_ Receptors and Cognition

While preclinical and clinical data suggest that activation of D_3_ receptors impairs cognitive processes and blockade or knock out of the receptors have pro-cognitive effects (Nakajima et al., 2013), much less is known about the underlying mechanisms. Our data on hippocampal gamma oscillations may offer an explanation. The hippocampus, expressing D_3_ receptors (Bouthenet et al., 1991; Richtand et al., 1995; Khan et al., 1998), is involved in a line of cognitive cues also affected by D_3_ receptors (Rubin et al., 2014). Moreover, gamma oscillations in the hippocampus play a key role in signal processing necessary for working memory, attention, learning and retrieval of memory by transiently enabling the communication between neurons and neuronal groups in different brain areas (Lisman and Jensen, 2013). In particular, the CA3 area of the hippocampus with its strong recurrent collateral associative connectivity is thought to keep information in the working memory by means of synchronous oscillation of pyramidal cells belonging to the assembly coding the pattern until it is stored in the synapses between CA3 pyramidal cells (Rolls, 2013). ACh release in the cortex closely follows the time-course of attention-demanding events (Parikh et al., 2007). Stronger gamma band modulations in V4 correlated with faster reaction times (Womelsdorf et al., 2006; Buehlmann and Deco, 2008) suggesting that the dynamic changes of gamma oscillations have behavioral consequences. Our results suggest that D_3_ receptor antagonists might exhibit their pro-cognitive effects by modulation of the dynamics of hippocampal gamma oscillations.

Dopaminergic neurons release DA within the forebrain in two different modes: the low tonic and the high phasic transmission mode. The tonic release underlies the background, steady state level of extracellular DA and is mediated by dopaminergic neuron population activity whereas the phasic release is produced by the activation of dopaminergic neuron firing by behaviorally relevant stimuli (Grace, 1991). High affinity D_3_ receptors may be activated during the tonic mode by the lower level of DA within the target sites, whereas low affinity receptors may only be activated by higher DA levels reached only during the phasic release. Our results suggest that the two release modes might trigger opposing effects and DA, by activating different receptors, may increase the signal-to-noise ratio: during the background release, gamma oscillations are inhibited by the high affinity D_3_ receptors. Salient stimuli may transiently increase DA levels in the forebrain activating low affinity DA receptors, such as the D_4_ receptor, which may increase gamma oscillations and thus cognitive processes such as attention, perceptual grouping, spatial navigation, and memory (Andersson et al., 2012).

### D_3_ Receptors and Schizophrenia

Besides genetic studies (Jönsson et al., 2003; Talkowski et al., 2006) there are also pharmacological evidences for the involvement of D_3_ receptors in schizophrenia. The atypical antipsychotic amisulpride is a pure D_2_ and D_3_ receptor antagonist with *K*i values of 1.7 and 2.5 nM, respectively (Tadori et al., 2011). Aripiprazole, another atypical antipsychotic, has a partial agonistic profile at both D_2_ and D_3_ receptors with *K*i values of 2.5 and 4.2 nM, respectively (Tadori et al., 2011). Because of the 7–40-fold higher affinity of endogenous DA for the D_3_ versus D_2_ receptors (Tadori et al., 2011), the D_3_ occupancy by these drugs might be lower (Girgis et al., 2011). Indeed, aripiprazole was only effective against cognitive impairment and negative symptoms at doses similar to or higher than its antipsychotic-like effective dose, suggesting that a higher D_3_ occupancy is needed for these effects (Adham et al., 2014). Cariprazine is a newly developed antipsychotic with profound pro-cognitive effects and very high affinity for the D_3_ receptor of 0.08 nM compared to the D_2_ receptors: 0.6 nM (Kiss et al., 2010). At a dose equivalent to the ED_50_ for antipsychotic like efficacy, cariprazine showed high levels (above 80%) of both D_2_ and D_3_ occupancy which may contribute to its better therapeutic outcome against cognitive and negative symptoms (Adham et al., 2014).

In individuals with schizophrenia, altered and instable gamma oscillations have been observed (Kwon et al., 1999). Our data suggest that antipsychotics with high affinity for the D_3_ receptor exert their antipsychotic effects by normalizing altered gamma oscillations. Since schizophrenia is characterized by disturbed (i.e., alternately enhanced *and* reduced) gamma oscillations, we suggest that partial agonism/antagonism at the D_3_ receptor may be a better therapeutic approach to treat schizophrenia than a pure antagonism. Partial agonists may stabilize gamma network activity at different circumstances such as different DA levels (Kiss et al., 2010; MacDonald and Bartolomé, 2010).

### Cellular Mechanisms Underlying the Inhibition of Gamma Oscillations in the CA3

We did not find significant differences between the effects of D_3_ receptors on ACh and KA-induced gamma oscillations (**Table 1**) as seen, e.g., for purinergic receptors (Schulz et al., 2012b). This may indicate that D_3_ receptors exert their non-specific inhibition of the gamma circuitries in the hippocampus by acting at a downstream target involved in both types of gamma oscillations. D_3_ receptors are G_i_ protein-coupled and decrease the intracellular level of cAMP (Missale et al., 1998). In the hippocampus they have been described on pyramidal cells (Khan et al., 1998; Swant et al., 2008). Hippocampal gamma oscillations are generated by synchronous rhythmic inhibition of pyramidal cell firing by fast spiking perisomatic parvalbumin^+^ interneurons (Gulyás et al., 2010). Their activation is due to excitatory drive supplied through feed forward or feedback excitatory inputs from granule and pyramidal cells, respectively. CA3 pyramidal cells fired APs at a rate of ∼10 Hz during gamma oscillations which was reduced by activation of D_3_ receptors to ∼6 Hz. Thus, within one gamma cycle in the presence of D_3_ agonists, less pyramidal cells fired. Moreover, the synchrony of the firing at a given phase of gamma oscillations was also reduced, suggesting that the coupling of APs to the gamma cycle became less accurate.

D_3_ receptors have been found to selectively downregulate T-type Ca^2+^ channels in the axon initial segment of auditory brainstem interneurons, which in turn reduced the AP output of these cells (Bender et al., 2010). Given the fact that axon initial segment Ca^2+^ transients have been observed also in pyramidal cells (Schiller et al., 1995), D_3_ receptors might also effectively inhibit the firing frequency and synchrony in the hippocampus by this mechanism. We also observed that D_3_ receptors inhibited membrane potential oscillations during gamma activity. These oscillations might reflect rhythmic synaptic inputs onto pyramidal cells, and their inhibition suggests that D_3_ receptors affect these inputs. Indeed, D_3_ receptor activation decreased the amplitude of inhibitory postsynaptic currents in CA1 pyramidal cells evoked in stratum radiatum possibly by causing endocytosis of GABA_A_ receptors in the apical dendrites (Swant et al., 2008).

## Conclusion

Fast network oscillations are the groundwork for cognitive processes such as attention, perceptual grouping, spatial navigation and memory. Moreover, disturbed gamma oscillations have been observed in diverse diseases such as schizophrenia, Alzheimer’s disease and autism. Our results show that activation of D_3_ receptors decreases power, coherence and dynamics of hippocampal gamma oscillations and provide a possible explanation how agonists may impair cognition and how antagonists exhibit pro-cognitive and antipsychotic effects. The complex network dynamics engendered by D_3_ receptor activation help shed further light on the generation and maintenance of gamma oscillations in the brain, and may one day be useful in developing targeted treatment options for a variety of neuropsychiatric conditions.

## Author Contributions

CL conducted and analyzed field potential recordings, SS and KH performed and analyzed intracellular recordings, RK had important role in interpreting the data, ZG designed and analyzed the experiments and drafted the manuscript, all authors revised the manuscript.

## Conflict of Interest Statement

The authors declare that the research was conducted in the absence of any commercial or financial relationships that could be construed as a potential conflict of interest.

## Abbreviations

5-HT_3_ receptor: 5-hydroxytryptamine 3 receptor
ACSF: artificial cerebrospinal fluid
AP: action potential
D_1_ receptor: dopamine D_1_ receptor
D_2_ receptor: dopamine D_2_ receptor
D_3_ receptor: dopamine D_3_ receptor
D_4_ receptor: dopamine D_4_ receptor
DA: dopamine
KA: kainate
Ki: dissociation constant
LFP: local field potential
Physo: physostigmine
